# Dynamic Impact of One-Year Integrated Rice–Crayfish Farming on Bacterioplankton Communities in Paddy Water

**DOI:** 10.3390/biology13121059

**Published:** 2024-12-17

**Authors:** Yiran Hou, Qiancheng Xu, Yanhong Yang, Rui Jia, Xiongjian Huang, Linjun Zhou, Bing Li, Jian Zhu

**Affiliations:** 1 Wuxi Fisheries College, Nanjing Agricultural University, Wuxi 214081, China; houyr@ffrc.cn (Y.H.); qianchengx@hotmail.com (Q.X.); jiar@ffrc.cn (R.J.); 2 Key Laboratory of Integrated Rice-Fish Farming Ecology, Freshwater Fisheries Research Center, Chinese Academy of Fishery Sciences, Ministry of Agriculture and Rural Affairs, Wuxi 214081, China; zhoulinjun@ffrc.cn; 3 Honghe Hani and Yi Autonomous Prefecture Fisheries Management Station, Mengzi 661100, China; yangyh_hani@hotmail.com (Y.Y.); huangxiongjian@hotmail.com (X.H.)

**Keywords:** integrated rice–crayfish farming, bacterioplankton community, paddy water, sustainable agricultural production

## Abstract

As global food security issues become increasingly severe, an important innovation in agricultural production patterns, namely integrated rice–fish farming, has been widely implemented around the world, especially in Asia. To assess the impact of integrated rice–crayfish (*Procambarus clarkii*) farming (IRCF) on agricultural ecosystems, we used Illumina high-throughput 16S rRNA gene sequencing to analyze differences in diversity, composition, co-occurrence network, and assembly process of planktonic bacterial communities in paddy water between traditional rice farming (TRM) and IRCF. Environmental factors and planktonic bacterial communities were evaluated during the tillering, jointing, flowering, and grain-filling stages on August 24, September 5, September 24, and October 16, respectively. Our findings revealed that, throughout the entire cultivation period, IRCF had no notable impacts on bacterioplankton community diversity in paddy water, but it changed the composition and relative abundance of the dominant bacterioplankton. Specifically, IRCF promoted the Chloroflexota during the tillering stage but reduced its presence during the grain-filling stage. It also significantly decreased the Bacillota during the jointing stage while notably enhancing Actinomycetota during the flowering stage. Furthermore, IRCF markedly improved the robustness and negative/positive cohesion within bacterioplankton co-occurrence networks during jointing and grain-filling stages. IRCF altered the assembly processes shaping planktonic bacterial communities, promoting a greater dominance of stochastic processes during the tillering, jointing, and flowering stages and a diminished dominance during the grain-filling stage. IRCF dramatically changed aquatic environmental factors, particularly during the jointing stage, by substantially increasing the TN, ammonium, nitrate, and phosphate levels in paddy water. These nutrient levels were closely correlated with the dynamics of the planktonic bacterial communities. Our findings underscore the considerable potential of IRCF in enhancing the stability of bacterioplankton communities and promoting rice growth while also providing valuable data and theoretical insights into the microbiological ecological impacts of IRCF on the agroecosystem.

## Simple Summary

To address global food security concerns, the integrated rice–fish farming system—an innovative approach in agriculture—has gained widespread adoption. However, research on its impact on agricultural ecosystems, particularly planktonic bacterial communities, is still very limited. This study analyzed the differences in diversity, composition, co-occurrence networks, and assembly processes of planktonic bacterial communities in paddy water between traditional rice monoculture and integrated rice–red swamp crayfish (*Procambarus clarkii*) farming during different rice growth stages. Our results revealed that an integrated rice–crayfish farming system significantly modified the bacterioplankton community structure, its co-occurrence networks and assembly dynamics, and environmental factors within paddy water in comparison to traditional rice monoculture. Importantly, these changes indicated the notable potential of integrated rice–crayfish farming in enhancing the stability of bacterioplankton communities and promoting rice growth. Our findings provided crucial data and theoretical insights into the microbiological and ecological impacts of integrated rice–crayfish farming on agricultural ecosystems, contributing to the sustainability and optimization of rice production patterns.

## 1. Introduction

The latest “The State of Food Security and Nutrition in the World 2024” report highlights that approximately 735 million individuals globally suffer from hunger, emphasizing the urgent need for solutions to food security [1]. Rice and aquatic products are crucial staples and providers of high-quality protein for the global population, respectively, and their production plays a vital role in maintaining global food security and stable supply [2,3,4,5]. Integrated rice–fish farming, as an important innovation in agricultural production patterns, is widely used globally, especially in Asia, and has now become an important approach to addressing the food crisis [6,7,8]. This agricultural production pattern synergistically combines rice cultivation and aquaculture, aiming to foster a mutualistic relationship between rice and aquatic animals within the limited water bodies of rice fields, thus achieving higher resource use efficiency and increasing food production [6,9,10,11,12]. Nevertheless, ecological research into integrated rice–fish farming remains considerably limited.

Microorganisms are indispensable indicators within agricultural ecosystems, providing a direct reflection of the ecological system’s state; meanwhile, due to their role in biogeochemical cycles, they directly or indirectly impact agricultural production [13,14,15,16,17,18]. Rice fields represent a complex habitat integrating aquatic and terrestrial elements, encompassing a complex micro-ecological network composed of soil and aquatic microorganisms [18,19]. Additionally, during agricultural production, environmental microbial communities can change in response to environmental alterations, biological factors derived from farmed animals, or interactions between the two [20,21,22,23,24]. Previous studies have shown that integrated rice–yellow catfish farming can considerably alter soil physicochemical properties, thereby affecting the bacterial communities in rice field soils and the production characteristics of the rice [25]. Furthermore, Hou, Jia, Sun, Li and Zhu [23] also identified varied effects and dynamic shifts in soil bacterial communities due to different stocking densities of red claw crayfish cultivation in rice fields. However, while most studies on integrated rice–fish farming have focused on soil microorganisms in rice paddies, the importance of microbes in paddy water has been largely overlooked. Indeed, planktonic bacterial communities are highly sensitive to changes in aquatic ecosystems caused by natural and anthropogenic pressures [26]. Agricultural activities have significantly altered the diversity and functionality of these bacterial communities [26,27]. Previous studies have shown that the introduction of farmed animals and the input of exogenous substances in agricultural production can affect bacterioplankton communities, which in turn impacts aquatic ecosystems [28,29,30,31]. Therefore, clarifying the dynamic effects of integrated rice–fish farming on bacterioplankton in rice paddy water is an important approach to understanding and assessing the ecological effects of this agricultural production pattern.

Integrated rice–red swamp crayfish (*Procambarus clarkii*) farming is the predominant practice in China’s integrated rice–fish farming [32]. In 2023, this agricultural production pattern spanned 1.69 million hectares, representing 56.37% of China’s total area dedicated to integrated rice–fish farming [32]. In practical rice production, the tillering stage, jointing stage, flowering stage, and grain-filling stage are very important growth and reproductive phases for rice [32,33]. Among them, the tillering stage is the critical period for determining the number of panicles [34]; the jointing stage is a period when significant changes occur both in the external morphology and internal physiology of rice, acting as a crucial link for ensuring high yield [35,36]; the flowering and grain-filling stages are key to determining the grain weight of rice, directly affecting the final yield and quality of the rice [34]. Hao, et al. [37] studied the impacts of integrated rice–red swamp crayfish farming on active organic carbon and microbial communities in the rhizosphere soil at different rice growth stages. However, research on the impact of integrated rice–crayfish farming on the planktonic bacterial communities in rice paddy water has not yet been reported.

In this study, we utilized Illumina high-throughput 16S rRNA gene sequencing to compare the impact of traditional rice cultivation and integrated rice–red swamp crayfish farming on the diversity, composition, co-occurrence networks, and assembly process of bacterioplankton communities in paddy water. Additionally, we explored the associations between environmental factors and bacterioplankton community dynamics. Our research aimed to understand the profound impact of integrated rice–red swamp crayfish farming on the agricultural ecosystem from the perspective of bacterioplankton community dynamics, contributing to sustainable agricultural production and optimizing rice production patterns.

## 2. Materials and Methods

### 2.1. Paddy Field Experiment and Sample Collection

**Figure 1:**
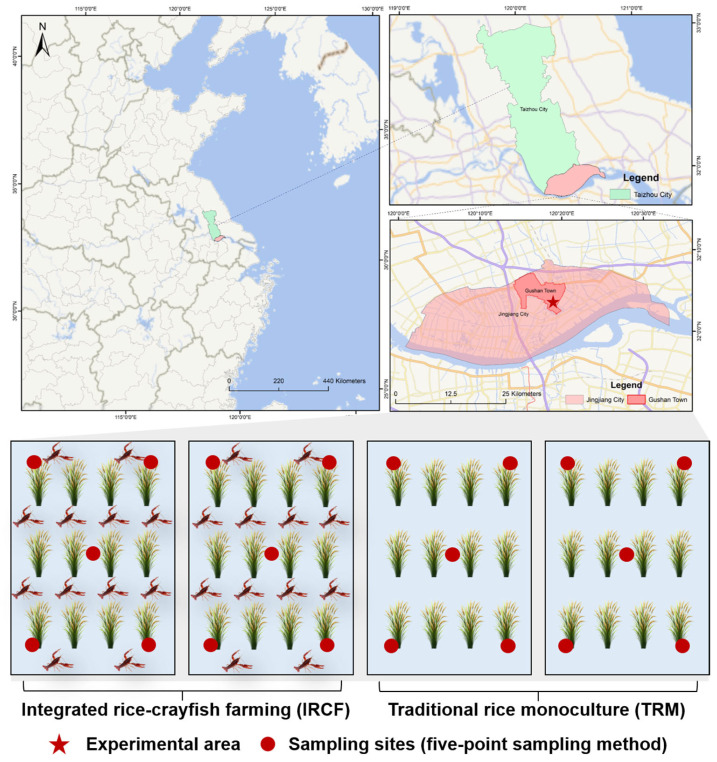
Schematic diagram of the experimental area.

This study was carried out in 2023 at the aquaculture facility of the Freshwater Fisheries Research Center, Chinese Academy of Fishery Sciences, situated in Jingjiang City, Jiangsu Province (32°5.515′ N, 120°19.947′ E, Figure 1). It involved four standard rice paddies, each spanning 4000 square meters. The experimental design included two groups: integrated rice–crayfish farming (IRCF) and traditional rice monoculture (TRM) (Figure 1). Rice planting occurred on 25 June, with a pre-sowing application of base fertilizer containing 16% nitrogen, 8% phosphorus, and 16% potassium at a rate of 0.03 kg/m^2^. Red swamp crayfish, *Procambarus clarkii,* was introduced on 16 July and harvested on 24 October, with an initial individual weight of 8.75 ± 0.48 g and a density of 2.5 individuals per m^2^ (approximately 21.88 g/m^2^). No pesticides were applied, and water was only added to offset evaporation without discharge during the experimental period. Red swamp crayfish were fed daily at 4:00 PM with commercial feed (constituting 1% of their body weight) from Cargill Feeds Co., Ltd. (Zhenjiang, China), which includes crude protein ≥ 40%, crude fat ≥ 5%, crude ash ≤ 16%, crude fiber ≤ 8%, total phosphorus ≥ 1%, moisture ≤ 12%, and lysine ≥ 2.2%. The temperature and pH of the paddy water varied from 23.13 to 29.65 °C and 7.24 to 7.89, respectively, while dissolved oxygen (DO) levels remained between 3.2 and 4.0 mg/L (Figure 1).

Water samples were collected from the rice paddy during the tillering, jointing, flowering, and grain-filling stages on 24 August, 5 September, 24 September, and 16 October, respectively [38]. For each rice paddy, the five-point sampling method was utilized to select five sampling sites, where 2.5 L of water was collected at each point (Figure 1). Ten sampling points were selected for each group, with 10 samples serving as the 10 replicates for each group. Following collection, the samples were filtered using 0.22 µm fiber filters and SHZ-D (III) circulating water-type multipurpose vacuum pumps (Shanghai Li Chen Bang Xi Instrument Technology Co., Ltd., Shanghai, China). Post-filtration, filters were removed using sterile tweezers and preserved in cryogenic tubes for subsequent analysis of plankton and bacterioplankton communities within paddy water. The physicochemical properties of the filtered water were determined.

### 2.2. Physicochemical Properties of Paddy Water

The temperature, pH, and DO of the paddy water were measured on-site at each sampling point using a portable multi-parameter analyzer (Hach HQ4300 Multi/ISE/3 channel, Hach Company, Loveland, CO, USA). The TN, TP, ammonia nitrogen, nitrite, and phosphate concentrations in paddy water were determined according to Chinese national standards by various spectrophotometric methods, as detailed in Table 1. Before measuring each batch of samples, calibration curves were measured following the same operational procedures as those used for the samples (Table 1). Calibration curves were plotted in real time, with six measurement points on each curve and a correlation coefficient (r) greater than 0.999.

### 2.3. DNA Extraction, Sequencing, and Data Processing

DNA from bacterioplankton in paddy water samples was extracted by the E.Z.N.A.^®^ Water DNA Kit (Omega Bio-tek, Norcross, GA, USA). Following quality assessment and quantification, the V3-V4 regions of the 16S rRNA gene in planktonic bacteria were amplified with the primers 341F (5′-CCTAYGGGRBGCASCAG-3′) and 806R (5′-GGACTACNNGGGTATCTAAT-3′). Quantification was performed using the Quantus™ Fluorometer (Promega, Madison, WI, USA). The resulting DNA products were pooled for library construction, and the assembled amplicon libraries were subjected to paired-end sequencing on the Illumina NovaSeq PE250 platform (Shanghai BIOZERON Co., Ltd., Shanghai, China) according to standard protocols.

The original sequencing reads were subjected to quality control using FASTP (version 0.18.0) and assembled with FLASH (version 1.2.11), setting the minimum overlap length to 10 bp and allowing a maximum mismatch error rate of 2% [44,45]. Subsequently, the remaining reads were processed to remove repeated sequences and analyzed using the Divisive Amplicon Denoising Algorithm 2 (DADA2) in Quantitative Insights into Microbial Ecology 2 (QIIME 2) to identify insertions/deletions and substitutions, classifying them as Amplicon Sequence Variants (ASVs) [46]. Paired reads were trimmed and filtered, allowing a maximum of two expected errors per read (maxEE ≤ 2). The planktonic bacterial ASVs were classified and identified using the RDP classifier software (version 2.2) through the Silva (SSU132) database [47].

### 2.4. Statistical Analysis

Our research utilized Shannon, Simpson, Chao1, Pielou_J, and Sørensen–Dice coefficient indices to evaluate the diversity, richness, and evenness of planktonic bacterial communities in paddy water. We computed the Bray–Curtis distances and assessed differences in these communities across various periods and groups via Principal Coordinates Analysis (PCoA) coupled with the Permutational Multivariate Analysis of Variance (PERMANOVA) test. Furthermore, we conducted independent samples *t*-tests to investigate the differences in environmental variables and bacterial community diversity between the IRCF and TRM groups during each specific cultivation stage. Additionally, the Wilcoxon rank-sum test was used to analyze differences in the composition of planktonic bacterial communities between the IRCF and TRM groups during each designated period. The correlations between water environmental conditions and the planktonic bacterial communities, as well as the relative contributions of water environmental conditions to changes in planktonic bacterial communities, were assessed using distance-based redundancy analysis (db-RDA), linear regression, and aggregated boosted tree (ABT) [48,49,50]. Based on the Spearman correlation matrices (Spearman r > 0.6 and *p*-value < 0.05) of the bacterioplankton community sequencing data, the co-occurrence network was established, and robustness and negative/positive cohesion values were calculated [51]. Differences in the robustness and negative/positive cohesion values were addressed through top removal by Tukey’s Honestly Significant Difference (Tuke’s HSD) analysis. In addition, the Neutral Community Model (NCM) was used to assess the relative contributions of deterministic and stochastic processes to the assembly of microbial communities in pond water [52].

## 3. Results

### 3.1. Dynamics of the Environmental Variables

**Figure 2:**
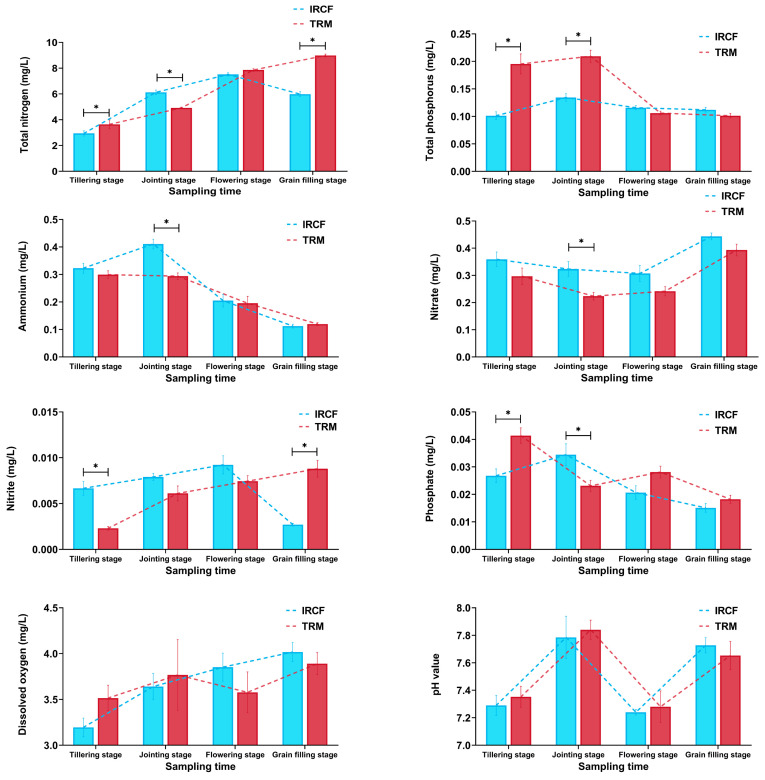
Dynamic changes in the physicochemical properties of paddy water across the IRCF and TRM groups throughout the entire cultivation period. An asterisk indicates significant differences between the IRCF and TRM groups at specific cultivation stages (*p* < 0.05).

The dynamic changes in the physicochemical properties of paddy water across all the groups are shown in Figure 1. The obvious impacts of IRCF on the physicochemical properties of paddy water were mainly manifested during the rice tillering stage and jointing stage (Figure 2, *p* < 0.05). At the tillering stage, compared to the TRM group, IRCF considerably reduced the contents of TN, TP, and phosphate in the paddy water but significantly increased the nitrite level (Figure 2, *p* < 0.05). During the jointing stage, IRCF substantially promoted the TN, ammonium, nitrate, and phosphate concentrations in the paddy water but significantly decreased the TP level (Figure 2, *p* < 0.05). During the flowering stage and grain-filling stage, there were no remarkable differences between the IRCF and TRM groups in the TP, ammonium, nitrate, and phosphate concentrations in the paddy water (Figure 2, *p* > 0.05). However, IRCF clearly reduced the contents of TN and nitrite relative to the TRM group during the grain-filling stage (Figure 2, *p* < 0.05). Regarding the pH and DO values of the paddy water, no substantial variations between the IRCF and TRM groups were identified throughout the entire cultivation period (Figure 2, *p* > 0.05).

### 3.2. Dynamics of the Diversity and Composition of Bacterioplankton Communities

**Figure 3:**
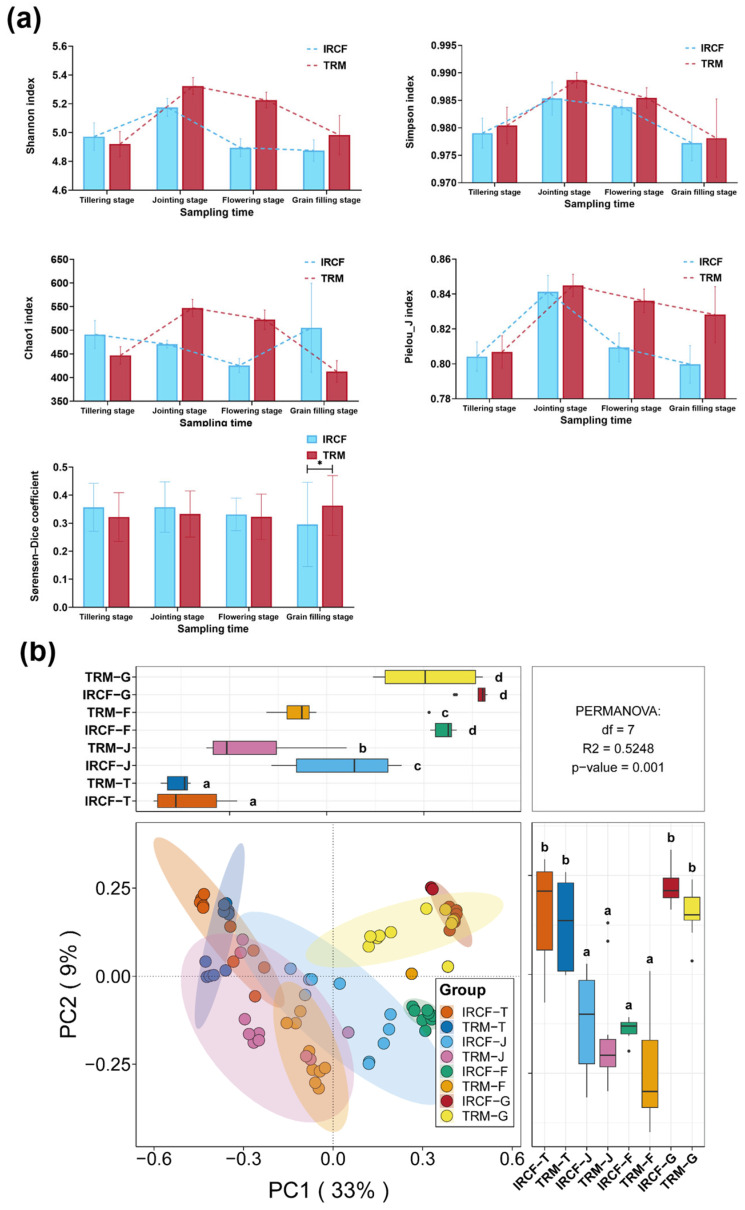
Diversity of bacterioplankton communities in paddy water across the IRCF and TRM groups. (**a**) Dynamic changes in the alpha diversity of bacterioplankton communities within paddy water across the IRCF and TRM groups during the entire cultivation period. An asterisk indicates significant differences between the IRCF and TRM groups at specific cultivation stages (*p* < 0.05). (**b**) Variations in bacterioplankton communities across different cultivation stages and groups were assessed using Principal Coordinates Analysis (PCoA) coupled with the Permutational Multivariate Analysis of Variance (PERMANOVA) test. Different lowercase letters indicate significant differences between different groups (*p* < 0.05).

Overall, throughout the entire cultivation cycle, the IRCF did not obviously impact the Shannon, Simpson, Chao1, and Pielou_J indices of bacterioplankton communities in rice paddy water compared to the TRM group (Figure 3a, *p* > 0.05). However, at the grain-filling stage, the bacterioplankton community within paddy water in the IRCF group exhibited a significantly lower Sørensen–Dice coefficient than the TRM group (Figure 3b, *p* < 0.05). Meanwhile, PCoA also revealed notable differences between the bacterioplankton communities at various stages and between groups (Figure 3b, *p* < 0.05). Notably, the bacterioplankton communities in IRCF and TRM groups were remarkably differentiated along the PC1 axis during the jointing and flowering stages (Figure 3b, *p* < 0.05).

**Figure 4:**
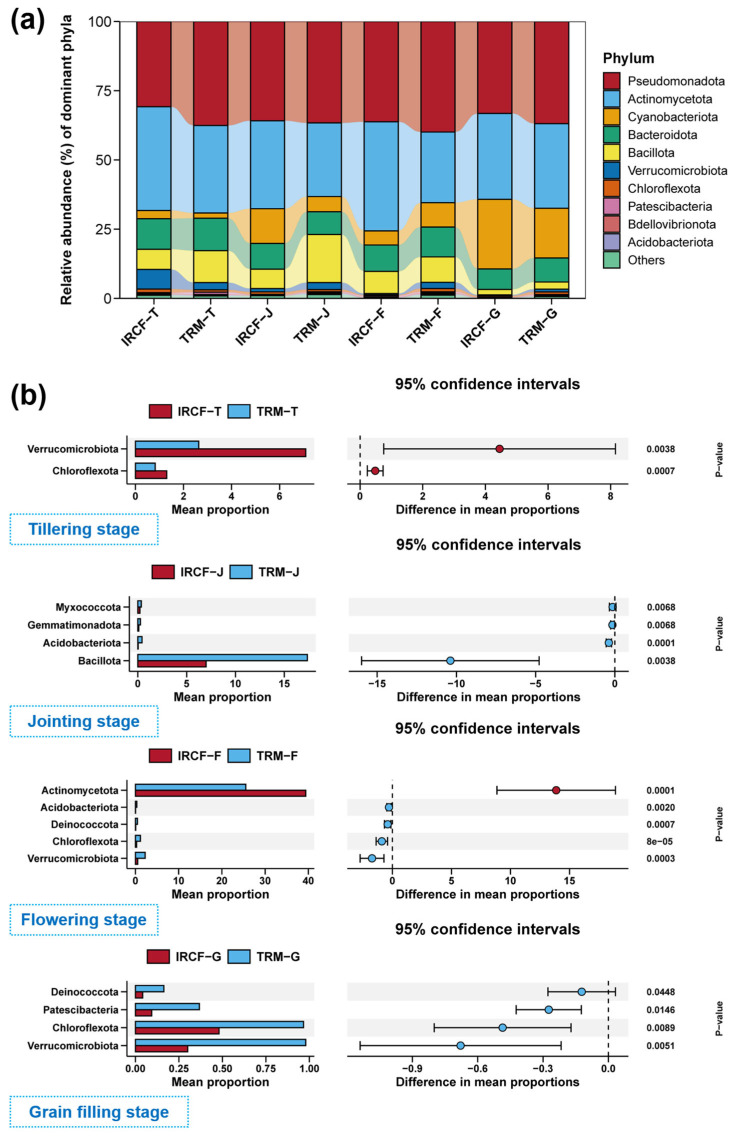
Dynamics of bacterioplankton community composition in paddy water across the IRCF and TRM groups. (**a**) Dominant phyla composition of the bacterioplankton communities within paddy water across the IRCF and TRM groups during the entire cultivation period. (**b**) Planktonic bacterial phyla with significant differences between the IRCF and TRM groups within rice paddy water during each specific cultivation stage.

In the rice paddy water, the dominant phyla (top ten by relative abundance) within the planktonic bacterial community predominantly comprised Pseudomonadota, Actinomycetota, Cyanobacteriota, Bacteroidota, Bacillota, Verrucomicrobiota, Chloroflexota, Patescibacteria, Bdellovibrionota, and Acidobacteriota (Figure 4a). The IRCF substantially influenced the composition of these planktonic bacterial communities at the phylum level (Figure 4b, *p* < 0.05). During the tillering stage, specifically, the IRCF group displayed considerably higher relative abundances of Verrucomicrobiota and Chloroflexota in comparison to the TRM group (Figure 4b, *p* < 0.05). In the jointing stage, the IRCF notably decreased the relative abundances of Bacillota and Acidobacteriota when compared to the IRM group (Figure 4b, *p* < 0.05). Throughout the flowering stage, relative to the TRM group, IRCF remarkably elevated the Actinomycetota level while diminishing the Acidobacteriota, Chloroflexota, and Verrucomicrobiota levels (Figure 4b, *p* < 0.05). Finally, during the grain-filling stage, the prevalence of Chloroflexota and Verrucomicrobiota within paddy water in the IRCF group remained markedly lower than that in the TRM group (Figure 4b, *p* < 0.05).

### 3.3. Dynamics of Co-Occurrence Networks of Bacterioplankton Communities

**Figure 5:**
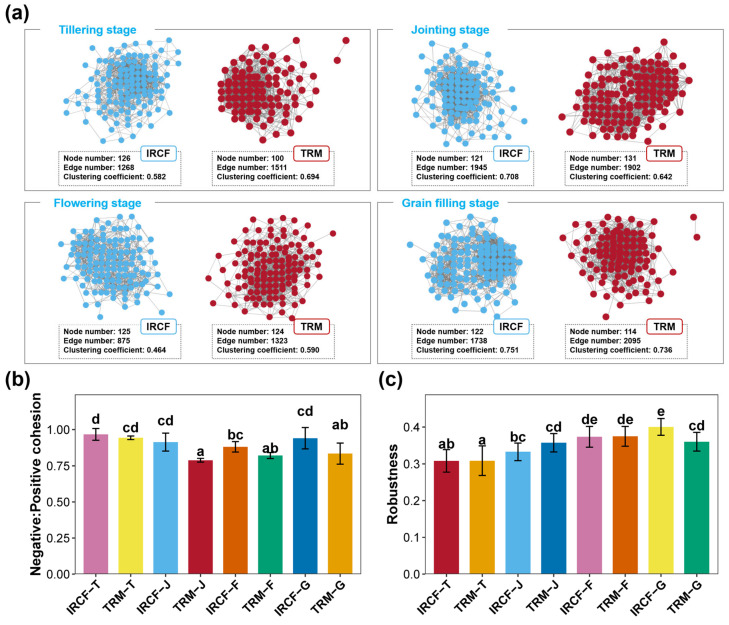
Dynamics of the co-occurrence network of bacterioplankton communities in paddy water across the IRCF and TRM groups. (**a**) The co-occurrence network of bacterioplankton communities and their topological parameters in the IRCF and TRM groups throughout the entire experimental period. (**b**) Differences in the negative/positive cohesion of bacterioplankton community co-occurrence network between the IRCF and TRM groups. (**c**) Differences in the robustness of bacterioplankton community co-occurrence networks between the IRCF and TRM groups. Different lowercase letters on the bar chart indicate significant differences between groups (*p* < 0.05).

Across the entire experimental period, IRCF markedly altered the co-occurrence networks of the planktonic bacterial community in paddy water compared to TRM (Figure 5). At the tillering stage, the co-occurrence network of planktonic bacteria in the IRCF group consisted of 126 nodes and 1268 edges, while that of the TRM group had 100 nodes and 1511 edges (Figure 5a). During the jointing stage, the co-occurrence network of the bacterioplankton community in the IRCF group comprised 121 nodes and 1945 edges, while that of the TRM group had 131 nodes and 1902 edges (Figure 5a). At the flowering stage, the IRCF group exhibited 125 nodes and 875 edges within the bacterioplankton community co-occurrence network, whereas the TRM group displayed 124 nodes and 1323 edges (Figure 5a). In the grain-filling stage, the co-occurrence network of the bacterioplankton community in the IRCF group featured 122 nodes and 1738 edges, as opposed to the TRM group, which had 114 nodes and 2095 edges (Figure 5a). Additionally, during the jointing and grain-filling stages, the IRCF group considerably enhanced the negative/positive cohesion of the bacterioplankton community co-occurrence network compared to the TRM group (Figure 5b, *p* < 0.05). During the grain-filling stage, the robustness of the planktonic bacterial community co-occurrence network in the IRCF group was obviously higher than that of the TRM group (Figure 5c, *p* < 0.05).

### 3.4. Dynamics of the Assembly Processes Shaping the Bacterioplankton Community

**Figure 6:**
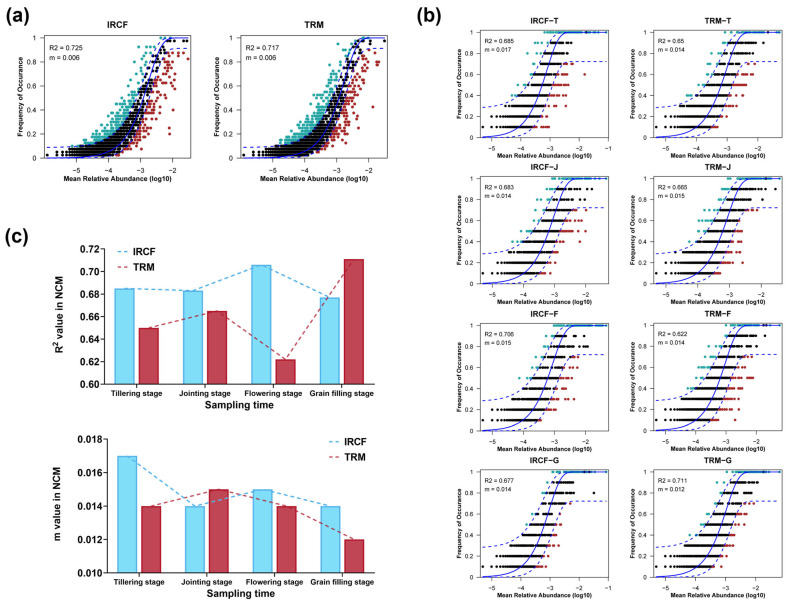
The assembly mechanisms of bacterioplankton communities in paddy water across the IRCF and TRM groups during the entire experimental period. (**a**) The overall assembly processes of the bacterioplankton communities in the IRCF and TRM groups. (**b**) The assembly processes of the bacterioplankton communities in the IRCF and TRM groups at each specific cultivation stage. (**c**) Dynamics of the R^2^ and m values in the neutral community model (NCM) evaluating assembly processes.

Throughout the entire cultivation period, whether in the IRCF or TRM groups, the R^2^ values of the NCM fitting results for the bacterioplankton communities in paddy water were all greater than 0.50, indicating that their shaping processes were predominantly driven by stochastic processes (Figure 6a). During the tillering, jointing, and flowering stages, the R^2^ values from the NCM fitting results of the bacterioplankton communities in the paddy water of the IRCF group were higher than those in the TRM group (Figure 6b,c). However, in the grain-filling stage, the R^2^ value from the NCM fitting result of the bacterioplankton community in the IRCF group was lower than that in the TRM group (Figure 6b,c). The m values from the NCM fitting results showed very small differences between the IRCF and TRM groups throughout the entire cultivation period (Figure 6b,c).

### 3.5. Associations Between Environmental Variables and Bacterioplankton Communities

**Figure 7:**
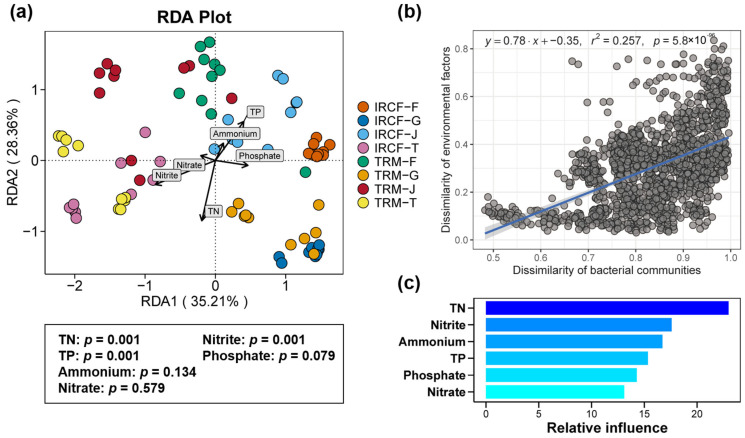
Associations between environmental variables and the bacterioplankton communities in paddy water. (**a**) Distance-based redundancy analysis (db-RDA) assessing the relationships between environmental variables and bacterioplankton communities in rice field water. (**b**) Linear regression identifying the intrinsic relationship between environmental factors and planktonic bacterial communities in rice field water. (**c**) Relative contributions of various environmental factors to the alterations in planktonic bacterial communities determined by aggregated boosted trees (ABT).

The results from db-RDA and linear regression demonstrated considerable correlations between planktonic bacterial communities and various environmental factors in rice field water (Figure 7a,b). Specifically, TN, TP, and nitrite were closely associated with these bacterial communities (Figure 7a, *p* < 0.05). In contrast, ammonium, nitrate, and phosphate did not exhibit remarkable correlations with bacterioplankton communities (Figure 7a, *p* > 0.05). Moreover, there was a significant relationship between the dissimilarity of environmental factors and the dissimilarity of bacterioplankton communities (Figure 7b, *p* < 0.05). According to ABT analysis, TN exerted the greatest influence on the variations observed in the bacterioplankton communities, followed by nitrite, ammonium, TP, phosphate, and nitrate (Figure 7c).

## 4. Discussion

### 4.1. Dynamics of Diversity and Composition of Bacterioplankton Communities in Paddy Water

Previous studies have shown that integrated rice–aquatic animal farming has a limited impact on the diversity of bacterial communities in paddy soil, but it significantly affects their composition [22,24,53,54,55,56]. Similarly, we observed analogous results in the bacterioplankton communities in paddy water. During the entire cultivation period, the IRCF had no significant impact on the diversity of the planktonic bacterial communities in the paddy water, but it changed the composition and relative abundances of the dominant bacteria. IRCF clearly promoted the phylum Chloroflexota during the tillering stage but reduced it during the grain-filling stage. Chloroflexota plays an important role in environmental carbon removal and participates in the nitrogen cycle [57]. Variations in the relative abundance of Chloroflexota induced by IRCF suggested functional differences in carbon and nitrogen cycling within the planktonic bacterial communities during the tillering and grain-filling stages.

Additionally, IRCF remarkably reduced the relative abundance of Bacillota during the jointing stage yet significantly promoted Actinomycetota during the flowering stage. Formerly known as Firmicutes, Bacillota plays a crucial role in nitrogen removal through its involvement in various biological denitrification processes, including nitrification–denitrification and anaerobic ammonium oxidation [58]. Actinomycetota, widely distributed globally, has members across aquatic and terrestrial ecosystems; they participate in the cycling of substances and degradation of complex polymers, possessing robust capabilities to remove organic and inorganic pollutants [59]. The suppression of Bacillota by IRCF might help reduce the competition for nitrogen sources between planktonic bacteria and rice; the promotion of Actinomycetota indicated an enhancement of the organic matter metabolic capacity of the planktonic bacterial communities in paddy water during the flowering period.

### 4.2. Dynamics of Co-Occurrence Networks and Assembly Processes of Bacterioplankton Communities in Paddy Water

In natural ecosystems, bacteria are interconnected rather than isolated, forming complex communities [60,61]. Co-occurrence networks among bacteria can reveal interconnection patterns within these communities, enhancing our understanding of their structure and characteristics [60,61,62]. Zhang, et al. [63] discovered that integrated rice–prawn (*Macrobrachium rosenbergii*) farming can substantially alter the co-occurrence network of soil bacterial communities, adversely affecting the stability of the soil bacterial community and the resilience of environmental variations. The integrated rice–fish and rice–crayfish farming can increase the number of edges and nodes in the co-occurrence network of soil bacteria, indicating enhanced stability of the bacterial community in paddy soil [25,53]. In the present study, we found that IRCF slightly affected the edge and node numbers within the co-occurrence networks of planktonic bacterial communities in paddy waters but markedly improved the robustness and negative/positive cohesion within these networks during jointing and grain-filling stages. Robustness and negative/positive cohesion represent the stability of the co-occurrence network; higher values indicate that the bacterial community is more stable [64,65,66,67,68]. Hence, the notable alterations in the co-occurrence network of bacterioplankton communities in paddy water revealed the significant promotive effect of IRCF on bacterioplankton community stability.

Deterministic processes and stochastic processes both simultaneously control the assembly of environmental bacterial communities [69]. In the NCM fitting, a higher R^2^ value indicates a closer match between the NCM and the actual bacterial community, suggesting a stronger influence of stochastic processes on the bacterial assembly [70,71]. In our study, stochastic processes dominated the assembly of the bacterioplankton community within paddy water throughout the entire cultivation period. Previous research has similarly shown that in agricultural and aquaculture ecosystems, the bacterioplankton assembly in water is primarily driven by stochastic processes [72,73,74,75]. Additionally, compared to traditional rice monoculture, the planktonic bacterial community in the IRCF group exhibited higher R^2^ values in NCM fitting during the tillering, jointing, and flowering stages, indicating enhanced dominance of stochastic processes. In contrast, a lower R^2^ value at the grain-filling stage suggests a diminished influence of these processes.

### 4.3. Dynamics of Environmental Factors and Their Associations with Bacterioplankton Communities in Paddy Water

Previous studies have shown that integrated rice–aquatic animal farming can alter the properties of paddy soil, thereby further impacting the soil microbial communities [22,24,25]. Similarly, in our study, IRCF dramatically changed aquatic environmental factors, particularly during the jointing stage, by substantially increasing the TN, ammonium, nitrate, and phosphate levels in paddy water. Similar to previous studies, the input of exogenous feed and the bioturbation by farmed animals in IRCF could be the primary factors affecting the environmental variables [23,55,76]. On the one hand, the red swamp crayfish, as a typical benthic animal, can remarkably promote material cycling at the water–soil interface and organic matter degradation in the soil, thereby enhancing the release of nutrients from the soil into the overlying water body [77,78,79]. On the other hand, the leftover bait and feces resulting from the input of external feed can serve as fertilizer for the rice crops in integrated rice–fish farming [76]. Meanwhile, it is also noteworthy that Zhang, et al. [80] found that the peak period for nitrogen uptake by rice occurs from the jointing to the flowering stages; Li, et al. [81] indicated that under normal phosphorus supply conditions, phosphorus accumulation during the rice jointing-booting stage contributes the most to rice yield. Hence, the enhancements in TN, ammonium, nitrate, and phosphate concentrations in the paddy water during the jointing stage in the current study might suggest the great potential of IRCF in promoting rice growth.

Environmental conditions can severely affect the environmental bacterial communities [82,83]. In this study, the nutrition levels within paddy water were closely related to the planktonic bacterial communities, particularly TN, nitrate, and TP. These nutrients critically affect bacterial communities by modifying growth, abundance, and activity, especially TN and TP, which play critical roles in shaping bacterial communities [84,85]. Additionally, the nitrate levels in the water body were also proven to be substantially correlated with bacterial communities with a profound impact [86]. Our study results also support these previous findings, with changes in the composition, co-occurrence networks, and assembly processes of bacterial communities in the IRCF group attributable to changes in environmental conditions such as TN, nitrate, and TP in the water bodies.

At the same time, we also found that the composition and diversity of the bacterioplankton community in paddy water fluctuated over time. Temperature is an important factor affecting the bacterioplankton community, and the response to water temperature is key to controlling the composition of the freshwater bacterioplankton community [87,88]. For example, higher temperatures can reduce the abundance and diversity of Cyanobacteriota [89]. The temporal changes in the composition and diversity of the bacterioplankton community, especially the increased relative abundance of Cyanobacteriota during the grain-filling stage, might be caused by differences in water temperature during the various growth stages of rice. Additionally, changes in nitrogen and phosphorus content over time might also be the other important reason for the temporal changes in the bacterioplankton community represented by Cyanobacteriota [90].

## 5. Conclusions

In summary, throughout the entire cultivation period, IRCF had no notable impact on bacterioplankton community diversity in paddy water, but it changed the composition and relative abundance of the dominant bacterioplankton; specifically, IRCF promoted the Chloroflexota during the tillering stage but reduced its presence during the grain-filling stage. It also significantly decreased the relative abundance of Bacillota during the jointing stage while notably enhancing Actinomycetota during the flowering stage. Furthermore, IRCF markedly improved the robustness and negative/positive cohesion within bacterioplankton co-occurrence networks during jointing and grain-filling stages, thereby contributing to a more stable bacterioplankton community. IRCF altered the assembly process shaping the planktonic bacterial community, promoting a greater dominance of stochastic processes during the tillering, jointing, and flowering stages and a diminished dominance during the grain-filling stage. IRCF dramatically changed aquatic environmental factors, particularly during the jointing stage, by substantially increasing the TN, ammonium, nitrate, and phosphate levels in paddy water. These nutrient levels were closely correlated with the dynamics of the planktonic bacterial communities, particularly the TN, nitrate, and TP concentrations. Our study highlighted the considerable potential of IRCF in enhancing the stability of bacterioplankton communities and promoting rice growth over a one-year period. However, due to the limitation of one year of data, it remains uncertain whether IRCF can have similar long-term effects on bacterioplankton communities. Therefore, future research will continue to focus on the long-term microbiological impacts of IRCF on the agroecosystem under different climatic conditions.

## Figures and Tables

**Table 1:** Methods for determining the physicochemical properties of paddy water.

Physicochemical Indicator	Measurement Methods
Total nitrogen (TN)	Alkaline potassium persulfate digestion—UV spectrophotometric method [39]
Total phosphorus (TP)	Ammonium molybdate spectrophotometric method [40]
ammonia	Nessler’s reagent spectrophotometry [41]
nitrate	Spectrophotometric method with phenol disulfonic acid [42]
nitrite	Spectrophotometric method with naphthalene ethylenediamine [43]
phosphate	Ammonium molybdate spectrophotometric method [40]

## Data Availability

The bacterial, fungal, and protist datasets supporting our study in this manuscript can be found in NCBI repositories. The accession number is PRJNA1188762.
